# Change in eating habits and physical activities before and during the COVID-19 pandemic in Hong Kong: a cross‐sectional study via random telephone survey

**DOI:** 10.1186/s12970-021-00431-7

**Published:** 2021-04-28

**Authors:** Jingxuan Wang, Eng Kiong Yeoh, Tony Ka Chun Yung, Martin Chi Sang Wong, Dong Dong, Xiao Chen, Maggie Ka Ying Chan, Eliza Lai Yi Wong, Yushan Wu, Zihao Guo, Yawen Wang, Shi Zhao, Ka Chun Chong

**Affiliations:** 1 School of Public Health and Primary Care, The Chinese University of Hong Kong, Hong Kong Special Administrative Region Hong Kong, China; 2 CUHK Institute of Health Equity, The Chinese University of Hong Kong, Hong Kong Special Administrative Region Hong Kong, China; 3 Centre for Health Systems and Policy Research, The Chinese University of Hong Kong, Hong Kong Special Administrative Region Hong Kong, China; 4 School of Public Health, Zhejiang University, Zhejiang, China; 5 School of Public Health, Li Ka Shing Faculty of Medicine, The University of Hong Kong, Hong Kong Special Administrative Region Hong Kong, China; 6 Shenzhen Research Institute, The Chinese University of Hong Kong, Shenzhen, China

**Keywords:** COVID-19, Diet, Nutrition, Physical activity, Social distancing, Mitigation, Sport, Public health

## Abstract

**Background:**

Hong Kong is a densely populated city with a low incidence and mortality of coronavirus disease 2019 (COVID-19). The city imposed different levels of social distancing including, the closure of sports venues and restrictions on eateries. This inevitably affects the eating behaviour and physical activities of the population. We examined the changes in eating behavior and physical activities before and during the COVID-19 pandemic, and identified sociodemographic factors associated with the behavioral changes.

**Methods:**

This was a cross-sectional study via a random telephone survey of Chinese adults conducted in Hong Kong from May to June, 2020 - a period in which social distancing measures were being imposed. We measured the physical activity habits from four aspects and dietary consumption patterns from seven aspects before and during the pandemic based on the World Health Organization’s guidelines and previous publications.

**Results:**

In total, 724 participants were recruited. Individuals were found to cook more frequently at home (*p* < 0.001) and order take-out (*p* < 0.001) during the COVID-19 pandemic. While no significant change in the frequency of fast food consumption was observed, we found significant increases in the frequency of eating fruits (*p* < 0.001) and vegetables (*p* = 0.004). The frequencies of walking, moderate-intensive sports, and high-intensity sports were significantly reduced (*p* < 0.001). We found that healthy lifestyle behaviors during the pandemic were negatively associated with participants’ economic status.

**Conclusions:**

Social distancing measures likely provided an opportunity for individuals to stay home and thus eat healthier. However, in a prolonged period of social restrictions, a lower physical activity level poses a risk to public health. Public health officials are thus advised to monitor physical health on a population-wide basis. The findings highlighted the importance of interventions tailored to individuals who have prolonged home stays - particularly for individuals in the low economic group.

## Background

First detected in December 2019 in the city of Wuhan, China, the novel coronavirus disease (COVID-19) is an infectious disease caused by the severe acute respiratory syndrome coronavirus 2 (SARS-CoV-2), which caused nearly 2.1 million deaths with more than 93.8 million infections in 218 countries as of January 16, 2021. Without population-wide administration of effective and safe vaccines, public health strategies such as social distancing and personal hygiene have been proved to be effective measures to control the pandemic. Considering the beneficial effects of physical activity and healthy dietary habits on health outcomes, several studies have examined the associations between the COVID-19 pandemic, its control measures, and people’s chronic health conditions. Control measures including stay-at-home orders, social distancing recommendations, and closures of parks/fitness rooms were found to be highly effective in reducing the diffusion of infections. However, these initiatives may limit outdoor activities, disrupt physical exercise routines, increase sedentary behavior, and encourage more time spent on electronic screens [1–4].

Several studies have investigated the influence of COVID-19 outbreaks on dietary changes [5, 6]. One speculation is that death and other negative events during the pandemic may lead to personal stress and anxiety, thus may induce consumption of alcohol and sugar-rich, energy-dense food [5]. In addition, driven by the anxiety of possible food shortages and the idea of minimizing unnecessary travel, people tend to purchase groceries with a longer shelf life that usually have high salt and fat content, while purchasing less fresh produce such as fruits and vegetables [6]. Physical inactivity has also been reported to contribute to a sedentary lifestyle with more snacks between meals or late at night [7]. However, some empirical studies have challenged the unidirectional impact of the pandemic [8, 9]. For instance, a large-scale Polish survey proposed two opposite patterns in lifestyle changes during the COVID-19 pandemic: “pro-healthy changes” in around 30 % of the population and “unhealthy changes” in 20 % of the population [9].

Multiple factors are associated with the pattern of behavioral changes, including the intensity of control measures (e.g., recommended vs. forced home office), society’s macroeconomic environment, and individuals’ age, employment status, family size as well as perceived health status [2, 10]. However, the effects of most factors on lifestyle behavior have been inconsistent among studies. Meanwhile, most empirical studies were conducted online, which may oversample younger individuals while neglecting the poor and elderly population with limited access to the internet.

With a population of 7.4 million and visitor arrivals of nearly six million/month in 2018 [11], the Hong Kong Special Administrative Region has been widely recognized as a tourism destination and international transportation hub. The large number of travelers arriving in Hong Kong posed challenges to infectious disease control. Following confirmation of the first COVID-19 case in Hong Kong on January 23, 2020, the number of confirmed COVID-19 cases increased to 9,452 on January 16, 2021. Throughout the pandemic, Hong Kong adopted a containment strategy of early identification and isolation of cases, with social distancing policies implemented in response to the extent of disease transmission. On 28 March 2020, catering businesses were required to stop selling food or drinks on sites, and public venues for sports and fitness centers were closed. The prohibition of group gatherings to no more than four people in public areas was announced on March 29, 2020 [12]. Thereafter, nightclubs and bars were also instructed to close from early April 2020, while working from home and flexible work arrangements were subsequently recommended [13]. Although these control measures were intermittently relaxed in response to changes in the epidemic, they have been re-imposed in response to surges of transmissions and are anticipated to largely impact people’s lifestyle including diet and physical exercise.

Hong Kong did not impose a complete lockdown for COVID-19 control, yet the city's healthcare system has not been overwhelmed. In spite of this, the social distancing measures such as restrictions on eateries and closure of sports facilities still disturb the behavior with regard to diet and physical activities. Many studies have shown that an unhealthy/imbalanced diet and physical inactivity increase the risk of obesity, weaken the immune system, increase sleep disorders as well as the prevalence of cardiovascular events, and are associated with a higher all-cause mortality rate [4, 6, 10]. The impact of public health strategies in response to the COVID-19 pandemic on people’s diet and physical activity patterns remains unclear. In this study, we aimed to examine the changes in eating habits and physical activities before and during the COVID-19 pandemic in Hong Kong, and to identify sociodemographic factors related to the behavior change.

## Methods

### Study design

A cross-sectional study using a random telephone survey was conducted from May to June, 2020, when social distancing measures were imposed. Random telephone numbers were generated from a directory of approximately 50 thousand household telephone numbers using a computer program. Telephone numbers were first selected from the database as seed numbers to minimize sampling errors. Another three sets of phone numbers were then generated using the randomization of the last two digits to include unlisted numbers. Duplicate numbers were then screened out. Telephone surveys were performed between 6 p.m. and 10 p.m. on weekdays by experienced interviewers. Upon successful contact with targeted households, one member of the household was selected from those family members using the last-birthday random selection method. Residents aged 18 or older were included in this study. Ethics approval was obtained before conducting the study and verbal consents were sought from all participants.

### Questionnaire design

The questionnaire consists of four sections:

i. Sociodemographics: Sex, age, marital status, education level, employment status, housing status, monthly household income level, and the number of children aged 2–18 years.
ii. Health-related information: Body weight, height, and medical history of chronic diseases (i.hypertension, diabetes, overweight/obesity, hyperlipidemia, fatty liver).
iii. Eating habits before and during the COVID-19 pandemic: Eating habits were measured by three widely used aspects of eating behaviors (i.e., *dining out*, *ordering take-away*, *cooking at home*) and four widely used aspects of dietary patterns (i.e. *consuming sugary drinks*, *consuming fast food*, *eating fruits*, *eating vegetables*), taking a published review article as reference [14]. We first investigated the participants’ perceptions on whether their behaviors with regard to each aspect had changed during the pandemic. We then asked them to complete seven pairs of items on behavior frequencies (times/week) before and during the pandemic—for example, “Before the pandemic, how many meals did you usually dine out per week?” and “During the pandemic, how many meals did you usually dine out per week?”
iv. Levels of physical activity before and during the COVID-19 pandemic: Physical activity was measured on four aspects (sitting/lying, walking, moderate-intensive sports, and high-intensive sports) based on World Health Organization’s guidelines [15]. For each type of exercise, participants were asked about the perceived changes, followed by open-ended questions regarding the total amount of time (minutes/week) spent on the exercise before and during the pandemic.

### Sample size determination

According to an international study on the same subject [16], assuming that 33.5 % of the population would have a decrease in the time spent in physical activities, a sample size of 700 is required to achieve a precision of 3.5 % for the estimate given a 5 % type I error. The sample size was also adequately powered to detect a significant difference in changes in the time spent on vigorous-/moderate-intensity exercise, walking, and sitting.

### Statistical analysis

Descriptive statistics were used to characterize the participants. The paired differences (Δ) in diet and exercise outcomes between the periods before and during COVID-19 were examined using paired t-tests. To identify the independent factors associated with the changes in diet and exercise outcomes before and during COVID-19, multiple linear regression analyses were conducted and regression coefficient (β) were presented. A p-value (*p*) < 0.05 was applied for statistical significance and all statistical tests were two-sided. We used SAS (version 9.4) to conduct the analysis.

## Results

A total of 724 participants were recruited for the study with a response rate of 59.1 %. Their characteristics are presented in Table 1. Of the 724 participants, 222 (30.7 %) were men and 502 (69.3 %) were women. The proportion of participants aged 64 years or above was 45.0 %, and 37.2 % of them were overweight. The majority (77.6 %) were married and 22.7 % had children aged 2–18 years. A quarter of the participants were employed full-time or part-time, 43 % lived in private permanent housing, and 21.6 % had household incomes of HKD30,000 (~ USD3,870) or above. Among them, 62.5 % did not have chronic diseases, while 30.9% and 11.7 % had hypertension and diabetes, respectively. Only 54.1 % self-rated their health as “good to very good.”

**Table 1:** Characteristics of participants (*N* = 724)

Characteristics	
Sex	
Male	222 (30.7)
Female	502 (69.3)
Age, years	
Below 64	398 (55.0)
64 or above	326 (45.0)
Weight, kg	57.4 (50.0-63.8)
Body mass index, kg/m^2^	22.2 (20.6–24.1)
Overweight (BMI > 23 kg/m^2^)	269 (37.2)
Not overweight (BMI ≤ 23 kg/m^2^)	455 (62.9)
Marital status	
Unmarried	162 (22.4)
Married	562 (77.6)
Having children aged 2 to 18	164 (22.7)
Occupation	
Full-time or part-time employed	182 (25.1)
Retired/student/unemployed	542 (74.9)
Income, HKD	
Below 30,000^a^	568 (78.5)
30,000 or above	156 (21.5)
Housing condition	
Public/rental housing	413 (57.0)
Private permanent housing	311 (43.0)
Education	
Secondary school or below	558 (77.1)
Tertiary education or above	166 (22.9)
Medical history of chronic conditions	
Hypertension	224 (30.9)
Diabetes	85 (11.7)
Overweight/Obesity	20 (2.8)
Hyperlipidemia	16 (2.2)
Fatty liver	4 (0.6)
No chronic diseases	450 (62.5)
Self-rated health	
Poor or fair	332 (45.9)
Good or very good	392 (54.1)

^a^ HKD30,000≈USD3,870

Statistics are expressed as n (%) and median (25^th^ percentile - 75^th^ percentile) for categorical and continuous variables respectively.

Table 2 presents the changes in diet and physical exercise habits during the COVID-19 pandemic. Because of the social distancing measures, there was a significant decrease in the frequency of dining out during the COVID-19 pandemic (Δ = -1.26 meals per week, *p* < 0.001). We also found that during the COVID-19 pandemic, the average weekly frequency of cooking at home (Δ = 1.06 meals per week, *p* < 0.001) as well as ordering takeaways (Δ = 0.48 meals per week, *p* < 0.001) increased significantly. The increase in the frequency of consuming sugary drinks was statistically significant, with a slight change of 0.03 times per week (*p* = 0.037), while there was no significant change in the frequency of fast food consumption. Unexpectedly, we found significant increases in the frequency of eating fruits (Δ = 0.14 times per week, *p* < 0.001) and vegetables (Δ = 0.07 times per week, *p* = 0.008) during the COVID-19 pandemic.

**Table 2:** Eating behavior and physical activities before and during the Coronavirus Disease 2019 (COVID-19) pandemic (*N* = 724)

	Before COVID-19	During COVID-19	Difference (95 % CI)	*p*-value
Dining out (meals/week)	2.38 ± 2.41	1.12 ± 2.06	-1.26 (-1.38, -1.14)	< 0.001
Ordering take-away (meals/week)	0.54 ± 1.36	1.02 ± 1.85	0.48 (0.39, 0.58)	< 0.001
Cooking at home (meals/week) ^a^	9.53 ± 5.30	10.59 ± 5.36	1.06 (0.89, 1.23)	< 0.001
Consuming sugary drinks (times/week)	3.54 ± 2.98	3.57 ± 2.99	0.03 (0.00, 0.06)	0.037
Consuming fast food (times/week)	0.40 ± 0.98	0.40 ± 1.08	0.00 (-0.04, 0.03)	0.940
Eating fruits (times/week)	8.50 ± 4.44	8.64 ± 4.42	0.14 (0.07, 0.21)	< 0.001
Eating vegetables (times/week)	9.96 ± 4.22	10.03 ± 4.24	0.07 (0.02, 0.12)	0.008
Going out for work (days/week)	1.3 ± 2.3	1.2 ± 2.1	-0.2 (-0.2, -0.1)	< 0.001
Sitting or lying (minutes/week)	1410.0 ± 851.5	1897.8 ± 973.7	487.9 (442.3, 533.5)	< 0.001
High-intensive sports (minutes/week) ^a, b^	155.4 ± 377.0	102.2 ± 309.3	-53.0 (-72.5, -33.6)	< 0.001
Moderate-intensive sports (minutes/week) ^b^	137.6 ± 283.3	108.5 ± 240.1	-29.2 (-41.9, -16.4)	< 0.001
Walking (minutes/week)	328.1 ± 312.5	236.4 ± 242.6	-91.7 (-110.8, -72.5)	< 0.001

*CI* Confidence interval. Statistics are expressed as mean ± standard deviation. *p*-value was obtained by a paired t-test for a comparison of frequency of eating behaviour and physical activities before and during the COVID-19 epidemic

^a^ One subjects refused to answer the habit of cook at home and two subjects refused to answer the habit of high-intensive sports

^b^ The definition of high and moderate intensive sports followed the *WHO’s Guidelines on Physical Activity and Sedentary Behaviour*

During the COVID-19 pandemic, there was a significant drop in the frequency of going out for work (*p* < 0.001). The frequency of sedentary behavior (i.e., sitting/lying) was significantly increased (Δ = 487.9 min per week, *p* < 0.001). In contrast, the frequencies of walking (Δ = -91.7 min per week, *p* < 0.001), moderate-intensive sports (Δ = -29.2 min per week, *p* < 0.001), and high-intensive sports (Δ = -53.0 min per week, *p* < 0.001) were found to be significantly reduced.

Tables 3 and 4 present the sociodemographic factors associated with the changes in eating habits and physical activities, respectively, before and during the COVID-19 pandemic. Male subjects and younger individuals (i.e., aged < 64 years) had a significantly larger increase in the changes in sugary drink consumption frequency with 0.07 times per week more than female (*p* < 0.05) and a 0.09 times per week more than the elderly (*p* < 0.05). Younger individuals were also significantly associated with a larger reduction in the frequency of dining out (*p* < 0.01) during the COVID-19 pandemic. Those with children had a significantly greater increase in the frequency of cooking at home than those without children (β = 0.55 meals per week, *p* < 0.01), which could be related to school closures and leaving children at home all day. Individuals reporting chronic condition(s) were less likely to perform moderate-intensive sports than those without any chronic conditions (β = -35.6 min per week, *p* < 0.05). Those who rated their health as “good or very good” were more likely to walk than those who rated their health as “poor or fair” (β = 48.84 min per week, *p* < 0.05).

**Table 3:** Regression coefficient (95 % confidence interval) of individual characteristics on the changes in eating behaviour before and during the COVID-19 pandemic

	Dinning out	Ordering take-away	Cooking at home	Consuming sugary drinks	Consuming fast food	Eating fruits	Eating vegetables
Male	-0.09 (-0.35, 0.17)	0.06 (-0.15, 0.27)	-0.12 (-0.50, 0.26)	0.07 (0.01, 0.13) *	0.06 (-0.02, 0.14)	-0.08 (-0.23, 0.07)	-0.01 (-0.13, 0.10)
Aged 64 years or above	0.44 (0.13, 0.74) **	-0.24 (-0.49, 0.01)	-0.27 (-0.72, 0.19)	-0.09 (-0.16, -0.02) *	0.00 (-0.10, 0.09)	-0.11 (-0.29, 0.06)	0.01 (-0.13, 0.14)
Overweight	0.00 (-0.24, 0.24)	0.08 (-0.11, 0.28)	-0.05 (-0.40, 0.31)	0.01 (-0.04, 0.07)	0.01 (-0.07, 0.08)	-0.01 (-0.15, 0.13)	-0.04 (-0.14, 0.07)
Married	0.01 (-0.27, 0.30)	-0.01 (-0.25, 0.22)	-0.37 (-0.80, 0.06)	-0.04 (-0.11, 0.03)	-0.03 (-0.12, 0.06)	-0.1 (-0.26, 0.07)	0.00 (-0.13, 0.13)
Having children	0.04 (-0.31, 0.38)	0.15 (-0.13, 0.43)	0.55 (0.04, 1.06) *	-0.03 (-0.11, 0.05)	-0.08 (-0.19, 0.03)	-0.14 (-0.34, 0.06)	-0.02 (-0.17, 0.14)
Without a full-time/part-time job	0.00 (-0.31, 0.32)	-0.16 (-0.41, 0.10)	0.03 (-0.43, 0.50)	0.11 (0.04, 0.19) **	0.05 (-0.05, 0.15)	-0.14 (-0.32, 0.05)	-0.01 (-0.15, 0.14)
Income < HKD30,000	0.34 (0.01, 0.68) *	-0.17 (-0.45, 0.10)	0.16 (-0.35, 0.66)	-0.05 (-0.13, 0.03)	-0.06 (-0.17, 0.04)	-0.36 (-0.56, -0.16) ***	-0.23 (-0.38, -0.07) **
Living in public/rental housing	0.20 (-0.05, 0.44)	0.07 (-0.13, 0.27)	-0.25 (-0.61, 0.11)	0.04 (-0.02, 0.10)	0.07 (-0.01, 0.14)	0.11 (-0.03, 0.26)	0.00 (-0.11, 0.11)
Tertiary education or above	-0.23 (-0.56, 0.10)	0.47 (0.20, 0.74) ***	-0.04 (-0.53, 0.45)	-0.07 (-0.15, 0.01)	0.05 (-0.06, 0.15)	-0.12 (-0.32, 0.07)	-0.08 (-0.23, 0.07)
Presence of chronic condition(s)	0.11 (-0.17, 0.38)	-0.09 (-0.31, 0.14)	0.03 (-0.39, 0.44)	-0.02 (-0.08, 0.05)	-0.07 (-0.15, 0.02)	0.05 (-0.11, 0.22)	0.04 (-0.09, 0.16)
Good or very good in self-rated health	0.02 (-0.23, 0.28)	-0.04 (-0.24, 0.17)	-0.12 (-0.50, 0.26)	0.04 (-0.02, 0.10)	-0.04 (-0.12, 0.05)	0.05 (-0.10, 0.20)	0.03 (-0.09, 0.14)

*p < 0.05; ** < *p* < 0.01; ***p < 0.001

The lower economic sector of the population, in terms of participants with lower income levels, non-full-time occupations, or lived in public/rental houses, adopted a healthy lifestyle to a lesser extent during the COVID-19 pandemic. Individuals without a full-time or part-time job had a larger increase in sugary drink consumption (β = 0.11 times per week, *p* < 0.01) compared to those who remained employed. When comparing before the COVID-19 pandemic, those with a lower monthly household income (i.e., < HKD30,000) dined out more frequently (β = 0.34 times per week, *p* < 0.05), and consumed less fruits (β = -0.36 times per week, *p* < 0.001) and vegetables (β = -0.23 times per week, *p* < 0.01) in their diet during the COVID-19 pandemic. In addition, we found that individuals without a full-time or part-time job had a larger increase in the time spent on sitting or lying down (β = 233.75 min per week, *p* < 0.001). Although those with a lower household income reported a greater change in frequency of walking (β = 79.33 min per week, *p* < 0.01), individuals living in public or rental housing had a smaller reduction in the time spent on high-intensive sports during the COVID-19 pandemic (β = -55.89 min per week, *p* < 0.01).

**Table 4:** Regression coefficient (95 % confidence interval) of individual characteristics on the changes in physical activities before and during the COVID-19 pandemic

	Going out for work	Sitting or lying	High-intensive sports	Moderate-intensive sports	Walking
Male	-0.02 (-0.13, 0.10)	-66.83 (-169.13, 35.48)	-25.02 (-68.62, 18.58)	-3.46 (-32.24, 25.33)	-21.09 (-64.26, 22.07)
Aged 64 years or above	0.06 (-0.08, 0.19)	-115.26 (-235.83, 5.31)	16.01 (-35.51, 67.53)	19.03 (-14.89, 52.96)	9.60 (-41.27, 60.47)
Overweight	0.03 (-0.08, 0.14)	-35.84 (-131.34, 59.65)	-2.42 (-43.13, 38.30)	0.30 (-26.57, 27.17)	12.23 (-28.06, 52.52)
Married	-0.03 (-0.15, 0.10)	-63.34 (-177.57, 50.89)	20.32 (-28.36, 69.01)	-5.56 (-37.7, 26.59)	-2.48 (-50.68, 45.71)
Having children	0.11 (-0.04, 0.27)	-4.49 (-141.61, 132.62)	47.47 (-10.98, 105.92)	23.05 (-15.53, 61.63)	40.82 (-17.03, 98.67)
Without a full-time/part-time job	0.52 (0.37, 0.66) ***	233.75 (108.41, 359.09) ***	39.95 (-13.47, 93.38)	-23.53 (-58.8, 11.74)	-43.03 (-95.92, 9.85)
Income < HKD30,000	-0.18 (-0.33, -0.03) *	-105.84 (-240.70, 29.02)	-15.99 (-73.47, 41.50)	7.78 (-30.16, 45.73)	79.33 (22.43, 136.23) **
Living in public/rental housing	0.03 (-0.08, 0.14)	83.65 (-13.75, 181.05)	-55.89 (-97.45, -14.34) **	12.45 (-14.96, 39.85)	-0.51 (-41.61, 40.58)
Tertiary education or above	-0.17 (-0.32, -0.02) *	-34.73 (-165.47, 96)	-44.17 (-99.89, 11.55)	-19.29 (-56.07, 17.50)	17.08 (-38.08, 72.24)
Presence of chronic condition(s)	0.11 (-0.01, 0.24)	23.03 (-87.57, 133.62)	9.56 (-37.71, 56.82)	-35.60 (-66.72, -4.48) *	-17.58 (-64.25, 29.08)
Good or very good in self-rated health	0.11 (0.00, 0.23)	30.91 (-70.59, 132.41)	-25.32 (-68.62, 17.98)	-18.87 (-47.43, 9.69)	48.84 (6.01, 91.66) *

*p < 0.05; ** < *p* < 0.01; ***p < 0.001

## Discussion

In response to the COVID-19 outbreak, many countries have imposed stringent measures to control the spread of the virus which may indirectly influence dietary and exercise habits. Hong Kong, a densely populated city with a low incidence and mortality of COVID-19, encountered different levels of social distancing measures, including the closure of sport venues as well as restricting the number of diners per table and the total number of hours that eateries were allowed to be open. While these stringent measures have mitigated the disease transmission in Hong Kong [12, 17], a successive number of outbreaks and the corresponding prolonged period of social distancing measures raised concerns about maintaining a healthy diet and physical activity. In our study, although we observed a significant reduction in the frequency of physical activity, it is notable that local residents consumed more fruits and vegetables, consistent with healthier eating behavior. In addition, we found that the population with a lower economic profile was more likely to be affected during the epidemic, having fewer healthier eating habits and doing less physical activity. Other sociodemographic factors such as sex and age were also associated with changes in diet during the epidemic.

While some studies indicated that eating patterns in various populations were unhealthier during the COVID-19 pandemic [16], we found that Hong Kong people increased their consumption of fruits and vegetables. The increased intake of healthy foods has seldom been reported in previous publications, with some exceptions in India [18], Thailand [2], and Australia [19], which found a decreased consumption of junk foods. As Hong Kong people have a fast pace of life, the adult population who tends to consume more food prepared in advance to save time, in particular, might be less aware of the nutritional information of their food and beverages, including cholesterol content [20]. In fact, a health behavior survey conducted in 2018/2019 revealed that about 96 % of the adult population had an inadequate daily intake of vegetables and fruits based on WHO’s recommendations (i.e., consuming ≥ 5 servings of vegetables and fruits per day) [21]. Given working-from-home recommendations and restrictions in eateries, in addition to the anxiety of being infected when dining out, individuals had more opportunity to cook at home, which enabled them to include more fruit and vegetables in their meals. This, on the other hand, reduced food consumption behavior in Hong Kong restaurants in which meals are generally regarded as “more meat, less vegetables.”

Similar to many studies [1, 2, 19], we found a significant reduction in physical activities in the local population during the pandemic. Since the start of the community outbreak, the Hong Kong government has closed all sports centers and venues. Together with the stay-at-home recommendation, the facilities and opportunities for physical activities were very limited for the public, and it is not surprising that almost all forms of physical activities were found to have significantly decreased. As indicated by the population survey before the pandemic [21], about 17 % of local adults already had insufficient physical activities based on the WHO’s recommendation (i.e., engaging ≥ 150 min per week of moderate-intensity or ≥ 75 min per week of vigorous-intensity physical activity). Provided a lengthy period of mitigation, the worsening impact on the sufficiency of physical activities poses a risk to public health, especially in the prevention of chronic disease in an aging population. Public health policies should therefore be tailored to promote appropriate physical activities at home. For example, public health officials in Thailand promoted the nationwide Fit from Home (FFH) campaign during the lockdown period [2]. The FFH campaign contained various promotional approaches to encourage a shift in outdoor physical activities to recommend home-based exercises, which were shown to be associated with an improvement in compliance with desirable physical activities.

In our study, populations in the lower economic group were observed to have a lower extent of balanced diet and exercise habits during the pandemic. The findings are consistent with some European studies in which low-income families tend to face more financial and social difficulties during large-scale social events and are more likely to adopt negative lifestyle changes [22]. For instance, with closed parks and public fitness rooms, residents in Hong Kong’s public rental housing, with an average living space per person of 10.4 m^2^ fewer than that of the general Hong Kong population, faced more difficulties in maintaining physical activity [23].

The study has several limitations. First, the health metrics of the participants such as body weight and medical history of chronic conditions were self-reported outcomes instead of being measured physically or confirmed clinically. This raises concerns about reliability and potential recall bias. Second, we did not consider variables on psychological health in the questionnaire design, and they may confound the relationships between behavioral health outcomes and the restrictions imposed. For instance, the stress of losing a job during the pandemic and the financial downturn may affect food consumption behavior. Third, our study only targeted the adult population, and it is expected that social distancing measures such as school closure would have a greater impact on children, particularly during their growth and development period [1]. We acknowledge that our resources did not enable us to conduct video interviews with children under parental guidance. Finally, the changes in lifestyle habits may be attributed to factors other than the COVID-19 pandemic, and a similar study conducted in cities with little impact conferred by COVID-19 may allow a more definitive conclusion.

## Conclusions

In conclusion, our study showed that social distancing measures likely provided an opportunity for the population to stay home and eat healthier; however, with a lengthy period of socio-economic restriction, the worsening impact on the adequacy of physical activities could pose a significant public health sequelae. Public health officials are thus advised to monitor physical health on a population-wide basis. Specifically, tailored interventions to promote healthy lifestyle are essential to enhance the well-being of the population groups affected by the pandemic.

## Data Availability

The datasets are available from the corresponding author on a reasonable request.

## Abbreviations

COVID-19: Coronavirus Disease 2019
SARS-CoV-2: severe acute respiratory syndrome coronavirus 2
FFH: Fit from Home
